# Editorial: Corrosion inhibition - recent advancements

**DOI:** 10.3389/fchem.2024.1493419

**Published:** 2024-09-25

**Authors:** Ashish Kumar, Abhinay Thakur, Eno E. Ebenso

**Affiliations:** ^1^ Nalanda College of Engineering, Bihar Engineering University, Science, Technology and Technical Education Department, Government of Bihar, Nalanda, India; ^2^ Division of Research and Development, Lovely Professional University, Phagwara, India; ^3^ Material Science Innovation and Modelling, North-West University, Mafikeng, South Africa

**Keywords:** corrosion, inhibitors, infrastructure, sustainability, electrochemistry, metals

Corrosion is a pervasive and insidious problem that affects a broad spectrum of industries, from oil and gas to transportation, construction, and beyond (Kaya et al., 2024). Chemical reactions among metals and alloys and their surroundings can cause metals and alloys to deteriorate over time, which can result in expensive restorations, downtime for operations, and even ecological catastrophes when essential infrastructure fails. For example, oil leaks can result from pipeline corrosion, and building and bridge structural steel deterioration can jeopardize structural integrity and safety. Corrosion has a huge financial impact; annual global expenses are projected to be in the trillions of dollars. Because of these risks, creating and using efficient corrosion inhibitors has become essential for protecting industrial assets and extending the life of materials (Shoair et al., 2024). The way corrosion inhibitors work is that they create a barrier of defense on the surface of metals, which stops or greatly slows down the electrochemical reactions that lead to corrosion. These inhibitors can be directly applied in situations where corrosion is a risk, as well as applied as coatings or fluid additives (Thakur et al., 2024). The quest for more effective, economical, and ecologically friendly solutions is the driving force behind the continuous research in corrosion inhibition.

This editorial summarizes the most recent developments in the sector, emphasizing creative strategies that support the larger objective of sustainable industrial practices while also improving the efficacy of corrosion inhibitors (Venkatachalam et al., 2024). For instance, Sulaimon et al. demonstrated the effectiveness of a modified okra-based polymer as a corrosion inhibitor for mild steel in a 1 M HCl solution. According to the findings, the grafted okra polymer considerably lessens corrosion; at 800 ppm, the inhibition efficiency reached 73.5%. Additional findings revealed that an approximate polymer concentration of 142.3 ppm, at a temperature of 60.4°C, and after 22.4 h of immersion could yield the greatest inhibitory efficiency of 88.2%.

Khan et al. compared the corrosion protection performance of neat epoxy and zinc phosphate (ZP) modified epoxy coatings on Al alloy 6,101 over a year. The study discovered that compared to clean epoxy coatings, ZP-modified epoxy coatings offered noticeably superior protection. In particular, ZP-modified coatings showed 70% lower corrosion rates and about 30% higher electrochemical resistance when compared to clean epoxy. Additionally, the ZP-modified coatings showed enhanced gloss retention and peeling resistance. The ZP coatings significantly reduced shrinkage and cracking throughout the physical aging test, demonstrating exceptional durability. According to the study, ZP pigments improve the protective qualities of epoxy coatings, which makes them better suited for long-term use in challenging conditions. Similarly, Rodríguez-Torres et al. investigated the corrosion inhibition of API 5L X52 pipeline steel in 0.5 M H_2_SO_4_ using *Tradescantia spathacea* extract. The extract inhibits corrosion with an optimal efficacy of 89% at 400 ppm concentration, as demonstrated by the results. At 60°C, however, its efficiency drops to 40%. According to the study, *T. spathacea* is a mixed-type inhibitor that affects both cathodic and anodic processes. When compared to untreated steel, scanning electron microscopy shows reduced damage on the metal surface, confirming the extract’s protective function.

Research conducted by Sanni et al. evaluated the effectiveness and economic viability of palm kernel shell extract as a corrosion inhibitor for thermo-mechanically treated steel in artificial seawater. At 500 ppm concentration, the extract showed a 98% inhibitory efficacy, efficiently adhering to the Langmuir adsorption isotherm and adsorbing onto the steel surface. The development of protective layers on the steel surface was validated by investigations using Fourier transform infrared spectroscopy and scanning electron microscopy showing the inhibitor to have financial and environmental advantages over conventional inhibitors. Thakur et al. explored the corrosion inhibition potential of Prinivil for mild steel in 1 M HCl solution using various experimental and computational techniques. According to the findings, Prinivil can suppress corrosion up to 97.35% of the time at 500 ppm. Prinivil’s efficacy is confirmed by the investigation using electrochemical, gravimetric, and scanning electron microscopy techniques; a high K_ads_ value and activation energy suggests a good adsorption affinity. Additional evidence that Prinivil produces a stable, protective coating on the steel surface comes from Monte Carlo simulations and molecular dynamics. All things considered, Prinivil shows to be a very successful corrosion inhibitor with strong theoretical and experimental findings.
